# Microbial Transformation of Chlordecone and Two Transformation Products Formed During *in situ* Chemical Reduction

**DOI:** 10.3389/fmicb.2021.742039

**Published:** 2021-11-04

**Authors:** Jennifer Hellal, Pierre-Loïc Saaidi, Sébastien Bristeau, Marc Crampon, Delphine Muselet, Oriane Della-Negra, Aourell Mauffret, Christophe Mouvet, Catherine Joulian

**Affiliations:** ^1^BRGM, Orléans, France; ^2^UMR 8030 Génomique Métabolique, CEA, Institut de Biologie François Jacob, Genoscope, Université d’Evry Val d’Essonne, Université Paris-Saclay, Evry, France

**Keywords:** chlordecone (CLD), West Indies, ISCR, bacteria, biotransformation

## Abstract

Chlordecone (CLD) is a very persistent synthetic organochlorine pesticide found in the French West Indies. Recently published work has demonstrated the potential of zero-valent iron to dechlorinate CLD by *in situ* chemical reduction (ISCR) in soils under water-saturated conditions, forming mono- to penta-dechlorinated CLD transformation products. These transformation products are more mobile than CLD and less toxic; however, nothing is known about their further degradation, although increasing evidence of CLD biodegradation by bacteria is being found. The present study began with the enrichment from wastewater sludge of a CLD-transforming community which was then inoculated into fresh media in the presence of either CLD or two of the main ISCR transformation products, 10-monohydroCLD (-1Cl-CLD) and tri-hydroCLD (-3Cl-CLD). Carried out in triplicate batches and incubated at 38°C under anoxic conditions and in the dark, the cultures were sampled regularly during 3 months and analyzed for CLD, -1Cl-CLD, -3Cl-CLD, and possible transformation products by gas chromatography coupled to mass spectrometry. All batches showed a decrease in the amended substrates (CLD or hydroCLD). CLD degradation occurred with concomitant formation of a nine-carbon compound (pentachloroindene) and two sulfur-containing transformation products (chlordecthiol, CLD-SH; methyl chlordecsulfide, CLD-SCH_3_), demonstrating competing transformation pathways. In contrast, -1Cl-CLD and -3Cl-CLD only underwent a sequential reductive sulfidation/S-methylation process resulting in -1Cl-CLD-SH and -1Cl-CLD-SCH_3_ on the one hand, and -3Cl-CLD-SH, -3Cl-CLD-SCH_3_ on the other hand. Some sulfur-containing transformation products have been reported previously with single bacterial strains, but never in the presence of a complex microbial community. At the end of the experiment, bacterial and archaeal populations were investigated by 16S rRNA gene amplicon sequencing. The observed diversity was mostly similar in the CLD and -1Cl-CLD conditions to the inoculum with a dominant archaea genus, *Methanobacterium*, and four OTU affiliated to bacteria, identified at the family (*Spirochaetaceae*) or genus level (*Desulfovibrio*, *Aminobacterium*, and *Soehngenia*). On the other hand, in the -3Cl-CLD condition, although the same OTU were found, *Clostridium* sensu stricto 7, *Candidatus Cloacimonas*, and *Proteiniphilum* were also present at > 2% sequences. Presence of methanogens and sulfate-reducing bacteria could contribute to sulfidation and S-methylation biotransformations. Overall, these results contribute to increasing our knowledge on the biodegradability of CLD and its transformation products, helping to progress toward effective remediation solutions.

## Introduction

Soils, surface water, and groundwater in Martinique and Guadeloupe (French West Indies, FWI) are contaminated with chlordecone [CLD; C_10_Cl_10_O or C_10_Cl_10_H_2_O_2_ in hydrated form; CAS number 143-50-0; decachlorooctahydro-1,3,2-metheno-2H-cyclobuta(c,d)pentalen-2-one], a very persistent synthetic organochlorine pesticide. CLD was widely used (3 kg⋅ha^–1^ per year; an estimated total of 300 t) in the FWI, officially from 1972 to 1978 and from 1981 to 1993, to protect banana plantations against the banana weevil (*Cosmopolites sordidus*). CLD is a highly hydrophobic organochlorine pesticide (Kow between 4.5 and 5.4), strongly adsorbed to the soil (log Koc = 4.1–4.2) and poorly soluble (2 mg⋅l^–1^ at 25°C) (UNEP, 2007; U.S. Environmental Protection Agency, 2012). In Martinique, 80% of soil analyses carried out on plots that had historically been cultivated with bananas between 1970 and 1993 (18,000 ha) show CLD contamination. Its persistence in the soils of the FWI has been estimated at several decades in nitisols, centuries in ferralsols, and half a millennium in andosols. CLD builds up in food chains, and long-term chronic exposure to CLD through food and drinking water can have serious consequences on human health. Indeed, CLD is a potential carcinogen and causes liver tumors in laboratory rats and mice (Reuber, 1979). It is a reproductive and developmental toxicant, an endocrine disruptor, and a neurotoxicant depending on the exposure doses (Dallaire et al., 2012; Boucher et al., 2013; Costet et al., 2015). It is also suspected of increasing the risk of preterm birth and delaying development in young children. Epidemiological studies have made it possible to link its chronic exposure to an increase in the occurrence of prostate cancer and its recurrence (Kadhel et al., 2014; Cordier et al., 2015; Emeville et al., 2015; Multigner et al., 2016; Brureau et al., 2019). Although preventive measures such as prior authorization for cultivation, the ban on fishing, the closure of aquaculture farms, or the control of foodstuffs on display have been installed to avoid transfer to local populations, pollution of the West Indian environment remains a major problem.

Following a study conducted by the French Geological Survey (BRGM, Colombano et al., 2009), a degradation treatment based on *in situ* chemical reduction (ISCR) was identified as a promising depollution technique to treat contaminated soils on a large scale. ISCR using zero-valent iron (ZVI) or nano-ZVI is a promising depollution method used for chlorinated organic pollutants in soils or contaminated aquifers (Li et al., 2021). From 2010 to 2016, a succession of projects tested this remediation method in the laboratory and in the field (Dictor et al., 2011; Belghit et al., 2015; Mouvet et al., 2017, 2020) and showed that ISCR leads to the formation of several dechlorination products, reducing the concentration of CLD by 70% in two of the three main types of West Indian soils (nitisols and ferralsols). This allowed plants to be cultivated with levels of contamination falling below the authorized maximum residue limit for CLD (20 μg⋅kg^−1^ wet weight) unlike untreated soils (Mouvet et al., 2017, 2020). The main degradation products formed during ISCR are hydrochlordecones that have lost one or more chlorine atoms, especially -1Cl-CLD (10-monohydrochlordecone) and -3Cl-CLD (trihydrochlordecone). Additional toxicity research has shown that these major transformation products are neither mutagenic nor genotoxic and have lower proangiogenic properties than CLD (Legeay et al., 2017). On the other hand, they are more mobile and can therefore reach groundwater more easily (Ollivier et al., 2020a, b). However, to date we do not have information on the biodegradability of these transformation products and still few elements on the biodegradation of the CLD parent molecule.

Despite the consensus at the time, which admitted that CLD was non-biodegradable (Cabidoche et al., 2009), Dolfing et al. (2012) argued that the bibliographic, thermodynamic, and experimental data suggested that the molecule is not completely refractory to a microbial attack under reducing conditions. Since then, with the progress made in analytical chemistry, several studies from independent laboratories have demonstrated a microbial degradation of CLD with opening of the bishomocubane cage and loss of a C_1_ fragment. In each case, CLD was incubated in laboratory conditions with either a bacterial consortia or a bacterial strain isolated from one of these consortia (i.e., *Citrobacter* sp. 86 and *Desulfovibrio* sp. 86) (Chaussonnerie et al., 2016; Chevallier et al., 2019; Lomheim et al., 2020). Recently, Della-Negra et al. (2020) have also shown that a change in incubation conditions of *Desulfovibrio* sp. 86 could also give rise to chlordecthiol, the sulfur analog of chlordecone alcohol. Taken together, these results have enabled to classify the identified transformation products resulting from CLD biotransformation into seven families. However, it should be noted that these experimental results were all obtained under artificial anaerobic conditions, which do not correspond to the real redox state of FWI soils. Additionally, several of these studies (Chevallier et al., 2019; Della-Negra et al., 2020; Lomheim et al., 2020) also highlight the presence of these seven families of transformation products in the West Indian environment, reviving the idea that the microbial biodegradation of CLD under natural conditions remains possible.

In the present study, the enrichment of a new microbial consortium from wastewater sludge capable of biotransforming 4 mg⋅l^–1^ of CLD within a few weeks under reductive laboratory-controlled conditions into the same nine-carbon compound (pentachloroindene, C_9_Cl_5_H_3_) as previously identified in Chevallier et al. (2019) is presented. Its ability to transform either CLD, -1Cl-CLD, or -3Cl-CLD, two of the major transformation products derived from ISCR, was established, and further characterization of the transformation products formed during the incubation reveal the sulfidation and possible methylation of the three parent molecules.

## Materials and Methods

### Chemical

CLD hydrate (98% purity) was purchased from LGC Standards (Molsheim, France). 10-Monohydrochlordecone (-1Cl-CLD) (97% purity) and trihydrochlordecone (-3Cl-CLD) (97% purity) were obtained from Alpha Chimica (Chatenay-Malabry, France). ^13^C_10_-chlordecone (CLD-^13^C_10_) at 100 mg⋅l^–1^ in nonane is commercialized by LGC Standards (Molsheim, France). 2,4,5,6,7-pentachloroindene C_9_Cl_5_H_3_, chlordecthiol, 10-monohydrochlordecthiols, and methyl chlordecsulfide were synthesized and purified according to the literature (Chevallier et al., 2019; Della-Negra et al., 2020). Chemical products used for microbiological media were purchased from VWR (France). Analytic-grade organic solvents (acetone, cyclohexane, and dichloromethane) were purchased from Fisher Scientific (Illkirch, France). The individual standards were prepared from solid standards dissolved in acetone (200 mg⋅l^–1^) and stored at −18°C.

### Consortium Enrichment

Before performing the experiment presented in this paper, enrichments were carried out using three samples: (1) sludge from a wastewater treatment plant (WWTP), collected in September 2014 from a settling tank at the Orléans-La-Source WWTP, (2) a nitisol soil in Martinique, collected in 2012, and (3) sediment from the Bourget lake, collected in 2012. The hypothesis behind using WWTP sludge and Bourget lake sediment was that bacteria found in these environments are likely to have been exposed to a large range of organic molecules, which could make them able to degrade CLD. Moreover, Orndorff and Colwell (1980) and Chaussonnerie et al. (2016) had previously isolated bacteria capable of transforming CLD from a WWTP. The hypothesis behind using soil from a contaminated field in Martinique was that bacteria exposed to CLD could be able to degrade it.

It took several years to enrich a CLD-transforming microbial community from these samples, following two steps. The first step lasted 32 months and consisted of fed batches set up in anaerobic conditions. The second step consisted of reinoculating the consortia in a liquid fresh medium. Fed batches consisted of either 150 ml of wastewater sludge and 50 ml of culture medium or 10 g of soil and 120 ml culture medium. The culture medium that was used is similar to the OECD 311 directive and composed of 2/3 Solution A (KH_2_PO_4_ 0.27 g⋅l^–1^; Na_2_HPO_4_⋅12H_2_O 1.12 g⋅l^–1^; NH_4_Cl 0.53 g⋅l^–1^, 1 ml of an oligo-element solution: SL9—see DSMZ 1257; pH 7) and 1/3 Solution B (CaCl_2_⋅2H_2_O 0.075 g⋅l^–1^; MgCl_2_⋅6H_2_O 0.1 g⋅l^–1^). Batches were supplemented with sodium acetate and sodium lactate (8 mM each) as a carbon source and Na_2_S and L-cysteine (0.4 mM each) to induce reductive conditions. Abiotic controls were established in the same conditions except that batches were autoclaved (121°C, 40 min) three times at 24-h intervals followed by the addition of sodium azide (200 mg⋅l^–1^). CLD was added to all batches in order to obtain concentrations of 2 mg⋅l^–1^ using a concentrated solution prepared in acetone. Finally, blank controls consisted only of culture medium supplemented with the carbon sources and reducing agents. All batches were flushed with nitrogen to eliminate O_2_ and incubated under agitation (80 rpm) in the dark at 30°C for 20 months and then at 38°C for the following 16 months. Temperature was raised as little degradation was observed at 30°C. During the first 32 months of enrichment, the batches were sampled (15 ml) and analyzed eight times (at months 0, 2, 7, 19, 20, 25, 28, and 32) to analyze chlordecone and its known degradation products by GC-MS (see below). At four of these sampling dates, T7, T20, T25, and T28, fresh culture medium, carbon sources, and CLD were added to the batches. When this occurred, GC-MS analyses were carried out before and after the feeding. Results from these fed batches showed an accumulation of CLD transformation product 2,4,5,6,7-pentachloroindene (C_9_Cl_5_H_3_), especially in the batches containing WWTP sludge compared to the controls and led us to conclude that a bacterial biotransformation of CLD had occurred.

Based on these results, it was decided to pursue to the second step of the enrichments using only the WWTP batches.

The second step was carried out to further enrich a CLD-transforming microbial community. This step consisted of reinoculating WWTP fed batches in fresh culture medium (as described for step 1 and supplemented with sodium acetate and sodium lactate, 4 mM each, and the same amounts of reducers) to confirm the production of C_9_Cl_5_H_3_. Finally, after four reinoculations, the CLD-transforming consortium was used to inoculate the kinetic experiment presented in the present paper and described below.

### Experimental Setup for the Kinetic Experiment

The experiment was carried out in 500-ml glass Schott bottles with PTFE hermetic caps (Q-Series Cap, GL45, Omnifit^®^) to minimize adsorption to materials and ensure anoxic conditions. Batches consisted of 460 ml fresh culture media (as described above) inoculated with 40 ml inoculum (around 8%) and supplemented with sodium acetate and sodium lactate (8 mM each) and 0.4 mM Na_2_S. Controls were identical except for the inoculum. Three conditions were set up in biotic and control batches: the first received CLD (condition 1), the second 10-monohydrochlordecone (-1Cl-CLD) (condition 2), and the third trihydrochlordecone (-3Cl-CLD) (condition 3). Initial concentrations of these substances were 4 mg⋅l^–1^ in the batches. All conditions for controls and biotic assays were carried out in triplicates. Samples were collected for analysis of transformation products, Eh measurements, and DNA extraction at the beginning of the experiment and then after 8, 19, 29, 43, 57, and 90 days. A further sample was collected after 8 months to elucidate the fate of -1Cl-CLD and -3Cl-CLD. As this was not sufficient to conclude, fresh transfer cultures were made of conditions 2 and 3 by inoculating them into fresh media in the same conditions as the initial experiment; these transfer experiments are further referred to as condition 4 (transfer of condition 2 with -1Cl-CLD) and condition 5 (transfer of condition 3 with -3Cl-CLD).

### GC-MS Methods

#### GC-MS Method 1

GC/MS/MS was achieved with a Bruker GC450 gas chromatograph, 1177 injector, automated sampler Combi Pal (CTC), and a triple-quadrupole mass spectrometer 300-MS. The instrument was equipped with a non-polar 30 m × 0.25 mm × 0.25 μm Rtx-1 column (Restek, Lisses, France) and a split/splitless injector. One microliter was injected in splitless mode. Mass spectrometry and a GC program have been described elsewhere (Bristeau et al., 2014).

### GC-MS Method 2

A Thermo Fisher Focus GC coupled to a single-quadrupole mass spectrometer (Thermo Fisher DSQ II) was used. The instrument was equipped with a non-polar 30 m × 0.25 mm × 0.25 μm DB-5MS column (Agilent J&W) and a split/splitless injector. Three microliters were injected in splitless mode. Ionization conditions and the GC program have been described elsewhere (Chevallier et al., 2019). Duplicate injections of all samples were performed.

### GC-MS Monitoring

#### Step 1 Enrichment Batches

The 15-ml samples were extracted with dichloromethane and then analyzed by GC-MS (*GC-MS method 1*), according to the following protocol: an internal standard (0.3 ml of 4 mg⋅l^–1^ CLD-^13^C in acetone) was first added to the sample to correct possible biases during extraction and analysis of CLD and its transformation products. After 6 h (at room temperature), 100 ml of dichloromethane was added and the flasks were shaken for 16 h on a ping-pong shaking table at a speed of 150 rpm. The samples were then centrifuged for 10 min at 2,500 rpm if necessary (in the presence of particles). Then, 10 ml of the organic phase was evaporated and transferred to cyclohexane for a final volume of around 1 ml. The extract was stored at –18°C before analysis by GC-MS. The recovery rates for this analysis protocol were estimated prior to the experiment and measured 103 ± 4% for CLD and 118 ± 2% for -3Cl-CLD.

Pentachloroindene was used as an identification standard. Its limited amount prevented us from developing a satisfactory quantification method. Instead, estimation of its relative abundance was provided by calculating the relative peak area of each chlorinated species (Mouvet et al., 2017).

#### Step 2 Batches and Kinetic Experiment

As there were no solid particles in the reinoculations or the batches set up to compare the transformability of CLD, -1Cl-CLD, and -3Cl-CLD, the extraction protocol was slightly modified: only 5 ml dichloromethane were added for 10 ml of culture medium. After 6 h agitation, 1.5 ml of organic phase were recovered and evaporated and the extract was retrieved in 1 ml cyclohexane. The recovery rates for this analysis protocol were estimated prior to the experiment and were 103 ± 5% for CLD, 74 ± 9% for -1Cl-CLD, and 72 ± 9% for -3Cl-CLD. As previously, C_9_Cl_5_H_3_ results are shown as the area of the peaks whereas -1Cl-CLD, -3Cl-CLD, and CLD are shown in percentage of the initial amount (for examples of chromatograms, see Supplementary Figure 1).

### Further Characterization of Transformation Products

The initial analytical protocols allowed us to demonstrate the appearance of pentachloroindene during the biodegradation of CLD. However, it did not permit to detect any other transformation products, e.g., lower chlorinated polychloroindene or hydrochlordecone analogs (-4Cl-CLD, -5Cl-CLD) in -1Cl-CLD and -3Cl-CLD conditions. A search for other transformation products was conducted using a protocol adapted from Chevallier et al. (2019) and Della-Negra et al. (2020). Sampling for the kinetic experiment and the transfer experiment was carried out after 8 months of incubation and 90 days of incubation, respectively. Two milliliters of each biotic and abiotic experiment were collected, acidified to pH 3 with HCl, and stored at –18°C. After defrosting, 750 μl of the aqueous samples were extracted three times with 250 μl dichloromethane. The combined organic layers were evaporated under a N_2_ flux, dissolved in 100 μl chloroform, and analyzed using *GC-MS method 2*. The unambiguous presence of chlordecthiol (CLD-SH), 10-monohydrochlordecthiols (-1Cl-CLD-SH), and methyl chordecsulfide (CLD-SCH_3_) was demonstrated in biotic incubations (Figure 2 and Supplementary Figures 2, 3). Identification was achieved using synthetic standards already available for these compounds (Della-Negra et al., 2020).

**FIGURE 1:**
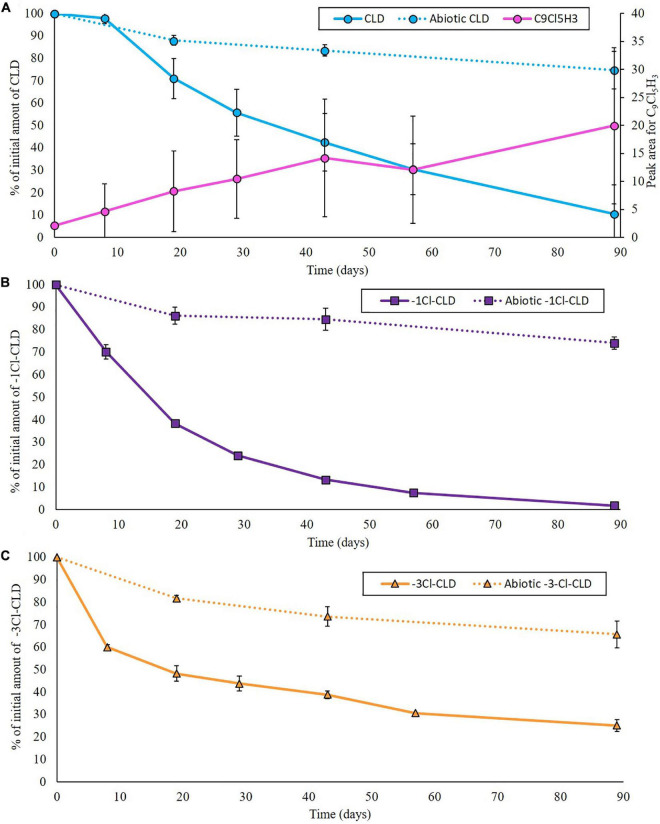
Evolution of CLD **(A)**, -1Cl-CLD **(B)**, and -3Cl-CLD **(C)** over time in biotic (full lines) and abiotic (dotted lines) incubations expressed as the remaining percentage in the solution and of the peak area measured for C_9_Cl_5_H_3_, which increased over time in the batches containing CLD. Data represent the average of three replicates, and error bars are the standard deviation.

**FIGURE 2:**
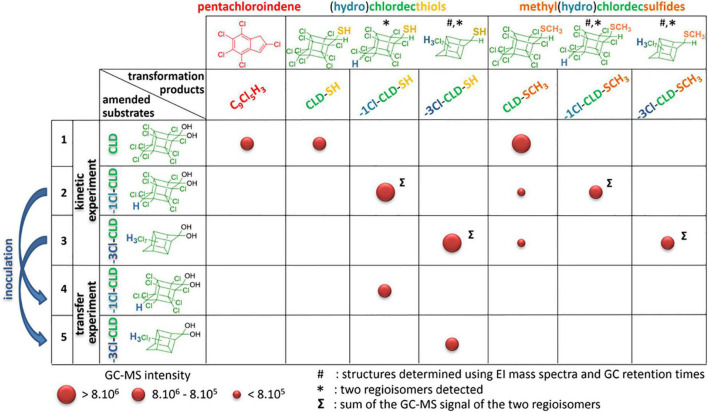
General overview of the transformation products resulting from CLD, -1Cl-CLD, and -3Cl-CLD incubations. Entries 1–3 correspond to the kinetic experiment conditions after 8 months while entries 4 and 5 refer to the transfer experiment (after 90 days). The size of the circles gives a qualitative overview of the intensity of the GC-MS signals observed (mean of two injections). Analyses were conducted using GC-MS method 2. Complete data are found in Supplementary Table 1.

The structure of the other transformation products resulting from the biotransformation of -1Cl-CLD and -3Cl-CLD were determined from their EI-mass spectra and their GC retention times (Supplementary Figures 3–7).

We first focused on the two signals barely separable at retention times 18.93 min and 19.03 min present in -3Cl-CLD conditions only (Figure 2 entries 3 and 5). They both shared the same EI mass spectrum (data not shown), thus suggesting a pair of diastereoisomers. Their two major isotopic patterns centered at m/z 167 and 202 strongly resemble the predominant EI fragments at m/z 201 and 236 both present in the -1Cl-CLD-SH and CLD-SH mass spectra, which were previously assigned to [C_5_Cl_3_SH_2_]^+^ and [C_5_Cl_4_SH_2_]^+•^ (Della-Negra et al., 2020). The observed downward shift of 34 units suggested the replacement of a Cl atom by an H atom in the studied structure. Due to the rather low intensity of the two GC-MS signals at 18.93 and 19.03 min, no expected molecular ion could be detected. Instead, the highest fragment centered at m/z 371 showed an isotopic distribution compatible with the ion [C_10_Cl_7_H_4_]^+^. The analogous ions, namely, [C_10_Cl_9_H_2_]^+^ and [C_10_Cl_10_H]^+^, present in -1Cl-CLD-SH and CLD-SH mass spectra, respectively, were indeed originated from the loss of HS by the molecular ions [C_10_Cl_9_H_3_S]^+•^ and [C_10_Cl_10_H_2_S]^+•^ (Della-Negra et al., 2020). Lastly, the retention times 18.93 and 19.03 min were significantly higher than that of starting material -3Cl-CLD (15.84 min) as in the case of both -1Cl-CLD-SH/-1Cl-CLD (22.89–22.97 min vs. 19.12 min) and CLD-SH/CLD (24.47 min vs. 20.46 min). Based on these observations and the context of the paralleled experiments carried out with CLD, -1Cl-CLD, and -3Cl-CLD, we assigned the trihydrochlordecthiol structure (-3Cl-CLD-SH; Figure 2) to these compounds.

We applied the same methodology to the two other couples of signals at retention times 24.33 min/24.45 min and 20.44 min/20.58 min formed in -1Cl-CLD and -3Cl-CLD conditions, respectively. The first two signals showed the exact same mass spectrum, again suggesting a pair of diastereoisomers. The three key isotopic patterns centered at m/z 203, 213, and 250 were identical to those visible in the mass spectrum of CLD-SCH_3_ (Supplementary Figure 7) and previously assigned to [C_5_Cl_4_H]^+^, [C_6_Cl_3_SH_4_]^+^, and C_6_Cl_4_SH_4_]^+^, respectively (Della-Negra et al., 2020). These ions strongly suggested the presence of a methylthioether moiety connected to the C5-fragment characteristic of the bishomocubane ring. An interesting difference with CLD-SCH_3_ lies in the absence of ion [C_5_Cl_5_]^+^ that was in favor of a lower chlorinated methylchlordecsulfide congener. Lastly, the highest isotopic pattern of the mass spectrum centered at m/z 488 matched perfectly with the simulation of the molecular ion [C_11_Cl_9_H_5_S]^+•^. We therefore ended up with the methyl monohydrochlordecsulfide structure for these transformation products (-1Cl-CLD-SCH_3_; Figure 2). Most of the previous features remained in the mass spectrum associated with the last couple of signals at 20.44 min/20.58 min (Supplementary Figure 4), except that they were shifted down by 34, 68, or 102 units, depending on the fragments (Supplementary Figure 7). We thus concluded a common methyl trihydrochlordecsulfide structure (-3Cl-CLD-SCH_3_; Figure 2).

### Microbial Community Monitoring

#### DNA Extraction

Ten milliliters of each microcosm were sampled at each sampling time and filtered through 0.22-μm sterile membrane filters (Ø 2.5 cm, Millipore, Bedford, MA, United States). Membranes were stored at −20°C until DNA extraction. DNA was extracted using the FastDNA^®^ Spin Kit for Soil and the FastPrep^®^ instrument according to the manufacturer’s instructions (MP Biomedicals, Santa Ana, CA, United States), with minor modifications (30 s lysis at a speed setting of 5.0 m⋅s^–1^, subsequent centrifugation of cell debris for 25 min). Extracted total DNA was quantified using the QuantiFluor dsDNA Sample Kit and the Quantus fluorimeter according to the manufacturer’s instructions (Promega, United States).

### Capillary Electrophoresis Single-Strand Conformation Polymorphism Fingerprinting

CE-SSCP (capillary electrophoresis single-strand conformation polymorphism) bacterial community fingerprinting was performed at each sampling date. The V3 region of the 16S rRNA gene was amplified by PCR from DNA extracts with forward primer w49 (5′-ACGGTCCAGACTCCTACGGG-3′) and 5′ FAM-labeled reverse primer w34 (5′-TTACCGCGGCTGCTGGCAC-3′), by 30-s hybridization at 61°C, and 30 s elongation at 72°C for 28 cycles as described previously (Delbès et al., 2001). One microliter of diluted PCR product (5- to 100-fold in nuclease-free water) was then added to a mixture of 18.6 μl of deionized formamide and 0.4 μl of GeneScan 600 LIZ internal DNA standard (Life Technologies, United States). To obtain single-strand DNA, samples were heat-denatured for 10 min at 95°C and immediately cooled on ice. CE-SSCP analyses were performed on an ABI Prism 310 genetic analyzer using a 47-cm-long capillary, a non-denaturing 5.6% CAP polymer (Life Technologies, United States), and the following electrophoresis conditions: run temperature 32°C, sample injection for 5 s at 15 kV, and data collection for 35 min at 12 kV. Alignment of the profiles using an internal DNA standard and assignment of peak positions were performed with Bionumerics software (Applied Maths, Sint-Martens-Latem, Belgium). Profiles are shown using the “gel view” option.

### Illumina Sequencing

At the end of the kinetic experiment (90 days of incubation), bacterial and archaeal diversity was determined by 16S rRNA gene metabarcoding. A portion of the 16S rRNA gene (V4-V5 region) was amplified using the barcoded, universal primer set (515WF/918WR) (Wang et al., 2009). PCR reactions were performed using the AccuStart II PCR ToughMix Kit, followed by cleaning (HighPrep PCR beads, MokaScience, La Madeleine Cedex, France). Pooled triplicates were submitted for sequencing on an Illumina MiSeq instrument at GeT-PlaGe (Auzeville, France). Fastq sequences were processed using FROGS (Escudié et al., 2018) based on the Galaxy analysis platform (Afgan et al., 2016). Sequences were demultiplexed and dereplicated, sequence quality was checked, oligonucleotides were removed from sequences, and sequences were filtered on additional criteria. Sequences were removed from the data set, if they exhibited ambiguous bases or did not match expectations in amplicon size. Remaining sequences were clustered into OTUs based on the iterative Swarm algorithm, then chimeras and singletons (OTUs containing only one sequence) were removed. Bacterial double affiliation was performed by blasting OTUs against the SILVA 138.1 database (Quast et al., 2013). OTUs with affiliation < 100% at the phylum level were removed from the data set. OTUs at lower taxonomic ranks than the phylum level were considered as “unidentified” below when the RDP bootstrap value was < 0.70. 16S rRNA gene amplicon sequencing data have been deposited in the European Nucleotide Archive (ENA) at EMBL-EBI under the study accession number PRJEB47148.

Diversity indexes (Chao1, Inverse Simpsons, and Shannon) were calculated based on the Illumina sequence data.

## Results

### Evolution of Chlordecone, -1Cl- Chlordecone, -3Cl- Chlordecone, and Redox Potential

Reductive conditions were maintained during the duration of the experiment between –150 and –250 mV due to the addition of Na_2_S (Supplementary Figure 8).

In the biotic incubations, concentrations of CLD, -1Cl-CLD, and -3Cl-CLD decreased over time (Figure 1). Less than 10% of the initially added CLD and -1Cl-CLD were found after 90 days of incubation whereas around 30% of -3Cl-CLD persisted in the batches. In the CLD conditions, a concomitant increase of C_9_Cl_5_H_3_ was observed through the increase in the size of the peak area (Figure 1). This degradation pathway with ring opening and dechlorination steps has been well documented for anaerobic microbiological degradation of CLD (Chaussonnerie et al., 2016; Chevallier et al., 2019; Della-Negra et al., 2020; Lomheim et al., 2020).

In abiotic conditions, the concentrations of CLD, -1Cl-CLD, and -3Cl-CLD decreased only slightly over time (Figure 1).

### Further Characterization of Transformation Products

All biotic incubations with CLD, -1Cl-CLD, and -3Cl-CLD led to a significant decrease in substrate concentration. This is most likely due to a microbiological transformation of all three compounds. In several past studies, the decrease of CLD could not be correlated with the formation of any transformation products and questions remained on its exact fate (degradation and/or sorption) (Sakakibara et al., 2011; Fernández-Bayo et al., 2013; Merlin et al., 2014; Amba Esegni et al., 2019). However, recent work by Lomheim et al. (2021) monitored the transformation of CLD into a suite of progressively more dechlorinated products, including a fully dechlorinated carboxylated indene product in anaerobic microcosms constructed with soil from Guadeloupe, suggesting that complete dechlorination of CLD is possible. For the endpoint sampling of the experiments (8 months), we therefore decided to change the extraction protocol and the analytical method used for monitoring. Acidification of the samples prior to extraction was applied as reported in Chevallier et al. (2019), and a longer gas chromatographic method was employed. GC-MS analysis revealed a series of polychlorinated compounds not only in -1Cl-CLD and -3Cl-CLD conditions but also for the CLD incubations. Comparison of the EI mass spectra and the GC retention times with the library of CLD transformation products that we have built over the years (Chaussonnerie et al., 2016; Chevallier et al., 2019; Della-Negra et al., 2020) allowed us to unambiguously identify CLD-SH, CLD-SCH_3_, and -1Cl-CLD-SH (as a pair of diastereoisomers). A more detailed analysis of the mass spectra of the other unknown compounds (see section “Further Characterization of Transformation Products”) enabled us to conclude the formation of -1Cl-CLD-SCH_3_, -3Cl-CLD-SH, and -3Cl-CLD-SCH_3_ (Figure 2, entries 2 and 3).

Additional LC-MS analysis did not allow us to detect any polychloroindenecarboxylic acids (data not shown). This series of polar transformation products also named as family C have been described in other CLD microbial degradation experiments (Chevallier et al., 2019; Lomheim et al., 2020, 2021). They differ from their polychloroindene congeners due to the presence of a carboxylic acid function that makes them only detectable using LC-MS instrumentation.

The presence of small peaks of CLD-SCH_3_ among the transformation products produced in the -1Cl-CLD and -3Cl-CLD was unexpected (Figure 2, entries 2 and 3). We hypothesized that this CLD-SCH_3_ came from the initial inoculum used (8% v/v in the kinetic experiment) that probably already contained CLD-SCH_3_ (not monitored at that time). To check this hypothesis, biotic incubations 2 and 3 were reinoculated into fresh medium (8% V/V), and after 90 days, GC-MS analysis showed only a production of -1Cl-CLD-SH and -3Cl-CLD-SH (entries 4 and 5; Figure 2 and Supplementary Figure 5) and no detectable amounts of CLD-SCH_3_, emphasizing that its presence in incubations 2 and 3 came from the initial inoculum.

### Evolution of Bacterial Diversity Fingerprint During the Incubation

CE-SSCP profiles of bacterial diversity obtained during the incubation showed that the CLD and -1Cl-CLD conditions (except replicate 3 for -1Cl-CLD) maintained a similar diversity fingerprint over time with two to three dominant bacterial strains and showed similarities with the inoculated consortium. On the contrary, the third replicate for -1Cl-CLD differed from the inoculum, evolving more toward the profiles found in the -3Cl-CLD condition where community fingerprints differed and suggested conditions which favored the development of a larger diversity (Supplementary Figure 9).

**FIGURE 3:**
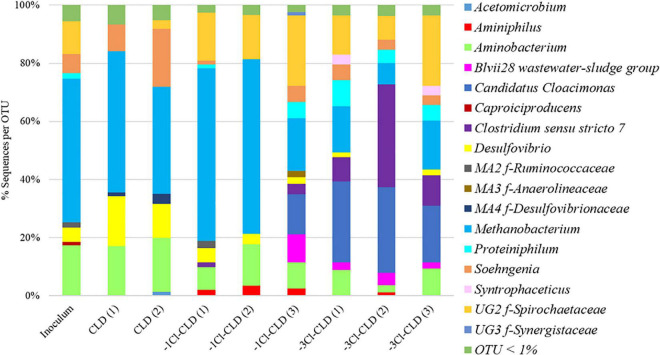
Histogram of the bacterial and archaeal genus represented by > 1% of the sequences and identified to the genus level. Multi-affiliated (MA) and unknown genera (UG) are given with their associated family (f).

At T6, after 90 days of incubation, bacterial and archaeal diversity was assessed with 16S rRNA gene metabarcoding (Figure 3). Results are consistent with the CE-SSCP diversity fingerprints with similar communities composed of three to four dominant bacterial OTUs in the inoculum, CLD, and -1Cl-CLD, belonging to the genera *Desulfovibrio*, *Soehngenia*, *Aminobacterium*, and to an unknown genus of *Spirochaetaceae.* Moreover, in the CLD and -1Cl-CLD [replicates (1) and (2)] and the inoculum, the main OTU that was found belongs to an archaeal genus, *Methanobacterium*, which represented up to 60% of the sequences. On the other hand, in the -3Cl-CLD condition and replicate (3) of the -1Cl-CLD condition, the relative abundance of this *Methanobacterium* is reduced and two other OTUs were present in significant relative abundances, *Candidatus Cloacimonas* and *Clostridium* as well as *Proteiniphilum*. It is possible that tolerance/resistance to CLD and -1Cl-CLD positively selected *Methanobacterium* whereas in less stressful conditions with -3Cl-CLD, other bacteria were more competitive.

**FIGURE 4:**
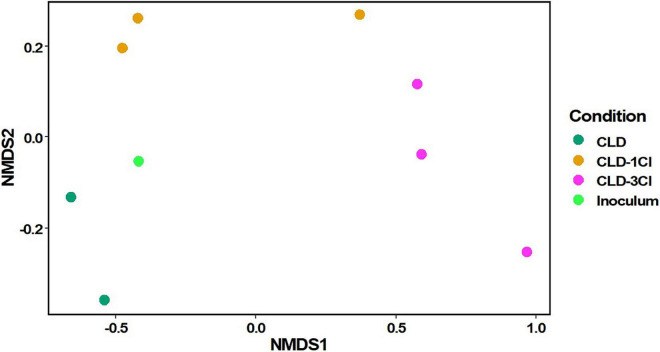
nMDS ordination plot of bacterial community diversity in the different incubation conditions and the inoculum.

An nMDS ordination (Figure 4), carried out on the OTU relative abundance data, highlights the similarities between the inoculum, CLD, and -1Cl-CLD [replicates (1) and (2)] and their difference with the -3Cl-CLD diversity. However, the ANOSIM test carried out on these data did not have significant differences between conditions (*p* > 0.001).

Finally, diversity indexes (Supplementary Figure 10) all show higher values for the -3Cl-CLD conditions, which corroborates the hypothesis that this condition was less toxic to the bacteria present in the consortium and that cells that had not grown in the inoculum, CLD, and -1Cl-CLD conditions were able to develop. Further work on the resistance/tolerance and toxicity of these compounds would be required to further understand this.

## Discussion

This work aimed to study the biodegradability potential of two major transformation products produced during ISCR CLD remediation in soils, namely, -1Cl-CLD and -3Cl-CLD, by a microbial consortium enriched from wastewater sludge and able to transform CLD into a nine-carbon compound (pentachloroindene, C_9_Cl_5_H_3_) and identify both the transformation products and the dominant genera involved.

There are only a few references of microbial transformation of CLD. Jablonski et al. (1996) is one of the earliest references with evidence of CLD biotransformation by the methanogenic archaeon *Methanosarcina thermophila* to polar and non-polar products using ^14^C-CLD. Other references testify to the formation of CLD derivatives in anaerobic or aerobic conditions (Fernández-Bayo et al., 2013; Merlin et al., 2014) but without detecting significant amounts of metabolites, as was found by Chaussonnerie et al. (2016) and Lomheim et al. (2021). During the 3-months-long incubation, CLD decreased concomitantly to the appearance of C_9_Cl_5_H_3_ (B family) as in the first enrichment steps. CLD has previously been shown to form C_9_Cl_5_H_3_ in similar conditions, in the presence of either bacterial consortia or isolated strains such as *Citrobacter* sp. 86 (Chaussonnerie et al., 2016) and *Desulfovibrio* sp. 86 (Della-Negra et al., 2020). Although the exact metabolic pathways have yet to be elucidated, recent work by Barbance et al. (2020) shows the involvement of cobalt-containing corrinoids in the microbial degradation of CLD, which could also be a possibility in the present study.

Since Chaussonnerie et al. (2016), several new families of degradation products derived from CLD have been identified by LC-HRMS (Chevallier et al., 2019; Lomheim et al., 2020, 2021). However, these families were not discovered at the end of the experiment in the present work (data not shown), suggesting either their further degradation in the incubation conditions or the inability of the enriched consortium to transform CLD to these compounds.

In the incubation conditions with either -1Cl-CLD or -3Cl-CLD, these compounds both appeared to be removed from the media. Further analysis of the transformation products did not reveal compounds from families B, C, D, and E (Chevallier et al., 2019); however, it did reveal the presence of sulfur derivatives, namely, thiols and methylthioethers (family F, Della-Negra et al., 2020). Reinoculation experiments (Figure 2, entries 4 and 5) led to an initial formation of thiols when the incubation period was shortened compared to the kinetic experiment, indicating a probable sequence mechanism of reductive sulfidation—S-methylation (Figure 2, entries 1–3). Reinvestigation of CLD biotic incubations using a modified analytical protocol showed the same type of sulfured transformation products (CLD-SH and CLD-SCH_3_). This proves that the present microbial enrichment can induce both the biodegradation of CLD (formation of pentachloroindene coming from the loss of carbon, oxygen, and chlorine atoms) and the biotransformation (conversion of the gem-diol to thiols and methylthioethers) of CLD, the two pathways being in competition in the applied incubation conditions. It is worth mentioning that neither -1Cl-CLD nor -3Cl-CLD appeared to be degraded, only biotransformed. This suggests that the position of the H atom(s) of -1Cl-CLD and -3Cl-CLD may inhibit the ring opening of the bishomocubane core, common to CLD and its two lower chlorinated congeners.

On the one hand, introduction of a sulfur atom on the chlordecone scaffolding has previously been demonstrated in presence of the sulfate-reducing bacterium *Desulfovibrio* sp. 86 (Della-Negra et al., 2020). To be effective, the biotransformation required an electron acceptor containing sulfur or hydrogen sulfide and a confined atmosphere. Microbiological reductive sulfidation is not limited to chlordecone since other carbonyl derivatives have been transformed into their corresponding thiol congeners using the same conditions with *Desulfovibrio* sp. 86 (Della-Negra et al., 2021). The authors suggested that two microbiological mechanisms may occur since aldehydes were converted during the growth phase while chlordecone, 10-monohydrochlordecone, and other ketones were biotransformed during the stationary phase. The present incubation conditions that fulfill the requirements reported by Della-Negra et al. (2021) (presence of H_2_S, confined atmosphere) and the presence of OTU affiliated to the sulfate-reducing genus *Desulfovibrio* are in favor of an analogous biotransformation. As in previous studies (Della-Negra et al., 2020, 2021), no transient thioketones could be detected. These intermediate compounds are observed in the chemical version of reductive sulfidation where a two-step process (initial S/O exchange followed by a reductive step) has been proven (Ozturk et al., 2010; Della-Negra et al., 2021). Moreover, the presence of OTU affiliated to the sulfate-reducing genus *Desulfovibrio* is in favor of this hypothesis in the present study.

On the other hand, while methylated thiols (methyl chlordecsulfides) were significantly formed after a switch in the incubation condition in the work of Della-Negra et al. (2020) using *Desulfovibrio* sp. 86, the present microbial community can induce both reductive sulfidation and methylation of the intermediate thiols in a one-pot manner. The absence of methyl chlordecsulfides in the transfer experiment after 90 days (Figure 2 entries 4 and 5; Supplementary Figure 5) and the systematic presence of both thiols and their methylated analogous in the kinetic experiment (Figure 2 entries 1–3; Supplementary Figures 2–5) are indicative of a probable sequential path. Another explanation would be the modification of the consortia contents, a hypothesis that is less probable as it would occur with both -1Cl-CLD and -3Cl-CLD substrates. In fermentative conditions, *Desulfovibrio* sp. 86 was able to methylate (Della-Negra et al., 2020), but no explanation has yet been proposed for this. In the present study, it is possible that the length of the experiment (8 months) enabled these reactions, although other explanations could exist.

Indeed, the OTU with the most relative sequence abundance (around 50%) was affiliated to *Methanobacterium*, a strictly anaerobic methane-producing archaeal genus that uses H_2_ as an electron donor. Further work is required to evidence the role of archaea in CLD biodegradation/biotransformation, but several hypotheses can be drawn. For instance, methylated-thiol-coenzyme M methyltransferase, a key enzyme involved in the methanogenesis (Ferry and Kastead, 2007), probably produced by this archaea in the present enrichment is capable of methylating thiols *via* an activated methylated corrinoid. A possible cometabolic methyl transferase activity *via* such methyl donor could take place. *Methanobacterium* could not only be involved in the methylation of CLD-SH and congeners but also induce the ring opening of the bishomocubane ring of CLD *via* the corrinoids it produced. Indeed, corrinoids, such as those required in methanogenesis, play a key role in the ring opening of CLD as demonstrated by *Citrobacter* sp. 86 mutants (Barbance et al., 2020). Cofactor F430 also required in the methanogenic metabolism was found to abiotically transform CLD in the presence of a reducing reagent (Jablonski et al., 1996). The dominance of *Methanobacterium* in incubations where CLD and -1Cl-CLD and -3Cl-CLD transformation products are being degraded is stressing that methanogenic conditions are adequate for the biotransformation of such complex molecules. This statement is in line with previously reported CLD biotransformation in such conditions by the methanogenic archaeon *Methanosarcina thermophila* (Jablonski et al., 1996). In the later study, CLD dehalogenation was suggested to occur while we rather expect sulfidation then methylation by *Methanobacterium*. Thus, our study strongly suggests that different pathways for CLD (and derivates) transformation might exist among methanogenic genera, such as sulfidation then methylation by the genus *Methanobacterium* found in our study or dehalogenation by the genus *Methanosarcina*, as suggested by Jablonski et al. (1996). This is further supported by the absence of a source on sulfur in the incubations with *Methanosarcina*.

Based on existing literature where, either from a thermodynamic point of view (Dolfing et al., 2012) or from an experimental point of view, there are no clear reasons why bacteria should not be able to respire CLD, reductive dehalogenation stands out as the best existing option to degrade CLD. However, while the redox potential in the present experiment was mostly comprised between –150 and –250 mV, no reductive dehalogenation, i.e., the direct replacement of a chlorine by a hydrogen atom, has been observed. This differs from the recent successful microbiological transformations of CLD that consistently reported the formation of hydrochlordecones, albeit in varying proportions. Indeed, only 10-monohydrochlordecone was reported as a minor transformation product by Chaussonnerie et al. (2016), Chevallier et al. (2019), and Della-Negra et al. (2020), while Lomheim et al. (2020, 2021) demonstrated the formation of a significant amount of several hydrochlordecones. However, in these studies the redox potential was not monitored so we cannot compare our experimental conditions to theirs.

In the work of Della-Negra et al. (2020), no formation of hydrochlordecones was observed when reductive sulfidation occurred. In the case of the single bacterium *Desulfovibrio* sp. 86, the observed inhibition of reductive dechlorination and ring opening dechlorination when reductive sulfidation takes place has been investigated (Della-Negra et al., 2020) and H_2_S formed by sulfate-reducing bacteria was shown to be responsible for such an inhibition. Using several biotic and abiotic additional experiments, the authors concluded that the corrinoids, known to be involved in CLD biodegradation, are probably deactivated due to the formation of a cobalt–sulfur covalent bond in the presence of H_2_S that accumulates in the confined atmosphere. Of course, the composition of the microbial consortia may also play a critical role.

Microbial diversity in the present experiment was lowest in the CLD and -1Cl-CLD conditions whereas it increased compared to the inoculum in the -3Cl-CLD condition and in one of the replicates (3) of the -1Cl-CLD incubations. Where diversity was lowest, it was dominated by known gram-negative genera (*Desulfovibrio*, *Aminobacterium*, *Soehngenia*, and *Spirochaetaceae*) and the archaeal *Methanobacterium* genus. Indeed, at high exposures of CLD, gram-negative bacteria have been shown to be more resistant to CLD (Mahaffey et al., 1982). To date, only gram-negative bacteria (*Pseudomonas aeruginosa* (Orndorff and Colwell, 1980), *P. putida*, *P. maltophilia*, *P. vesicularis* (George and Claxton, 1988), *Citrobacter* sp. 86, *Citrobacter* sp. 92 (Chaussonnerie et al., 2016), and *Desulfovibrio* sp. 86 (Della-Negra et al., 2020) as well as an archeon (*Methanosarcina thermophila*, Jablonski et al., 1996) have been reported to partially or completely transform CLD. All microbial degradations have been performed at elevated concentrations of CLD (equal or above 10 mg⋅l^–1^) that demonstrate a strong resistance of these microorganisms, which corroborates our observations. When diversity increased in our experiments, the representation of gram-positive genera increased (*Candidatus Cloacimonas, Clostridium*), possibly due to the reduced toxicity of -3Cl-CLD toward these bacteria, and the relative abundance of *Methanobacterium* and *Desulfovibrio* decreased, possibly explaining the incomplete transformation of -3Cl-CLD to -3Cl-CLD-SH and -3Cl-CLD-SCH_3_ in these incubations. Furthermore, the increase in the diversity indexes in -3Cl-CLD conditions also corroborates this hypothesis. Further work on the resistance/tolerance and toxicity of these compounds would be required to further understand this.

Identification of CLD transformation products and CLD-degrading microbial communities in soils and sediments of the French West Indies (FWI) demonstrates that natural transformation of CLD already takes place in these two types of environmental compartments (Chevallier et al., 2019; Della-Negra et al., 2020; Lomheim et al., 2020). Moreover, it has also been demonstrated that the microbial potential to further transform CLD with as many as nine chlorine atoms removed is present in these environmental compartments (Lomheim et al., 2020). However, more than 25 years after its use was forbidden, CLD pollution in the FWI is still a public health issue, implying that the mechanisms of natural CLD transformation are not particularly efficient. The present work is the fourth, after Orndorff and Colwell (1980), Chaussonnerie et al. (2016) and Lomheim et al. (2020), to report on enrichments from wastewater sludge capable of inducing CLD transformation. Anaerobic reactors of water treatment plants would thus appear as a suitable place to observe an even more effective CLD degradation. To date, only one study aimed at searching CLD transformation products in FWI raw wastewater (Devault et al., 2018). However, it failed to detect both CLD and the targeted transformation products (chlordecol and 8-monohydrochlordecone). Since sampling was carried out at the entry of the treatment plant and that chlordecol and 8-monohydrochlordecone have not been yet linked to microbial anaerobic transformation of CLD, these results should not discourage further investigation of this presumably efficient industrial degradation process.

Finally, we are aware that the experimental setup presented here is carried out in optimized laboratory conditions where Eh is maintained negative with very reducing conditions, theoretically favorable for dehalogenation. Thus, the conditions are not those found naturally in agricultural soils in the West Indies except when an ISCR treatment is applied (Mouvet et al., 2020) and the zero-valent iron combined with water saturation and soil compaction induces negative redox conditions and leads to the transformation of CLD to hydroCLDs such as the -1-Cl-CLD and -3-Cl-CLD used in this study and that would subsequently find themselves also in these conditions. In previous work, chlordecthiol (the S-containing derivative from CLD) was detected in mangrove sediments from Martinique Island (Della-Negra et al., 2020). This environmental compartment shows favorable conditions (sulfate-reducing bacteria and anoxic conditions) for the reductive sulfidation. One can imagine that S-methylation of such thiol would occur in the presence of the numerous sulfate-reducing microorganisms. Thus, we would expect to find similar several S-containing derivatives in the FWI indies environmental provided that ISCR treatment is to be set up and dedicated analytical methods are applied (no such additional analytical procedure was used in the work published by Mouvet et al. (2020).

## Data Availability Statement

The datasets presented in this study can be found in online repositories. The names of the repository/repositories and accession number(s) can be found below: https://www.ebi.ac.uk/ena/browser/view/PRJEB47148.

## Author Contributions

JH, CJ, AM, and MC designed the experiment. JH, DM, OD-N, P-LS, SB, and MC carried out the experiment and the analysis (chemical and microbial). JH, CJ, SB, MC, and P-LS analyzed the data. JH and P-LS drafted the manuscript. JH, AM, and CM carried out the funding acquisition. All authors have read and agreed to the published version of the manuscript, contributed to reviewing and editing the manuscript.

## Conflict of Interest

The authors declare that the research was conducted in the absence of any commercial or financial relationships that could be construed as a potential conflict of interest.

## Publisher’s Note

All claims expressed in this article are solely those of the authors and do not necessarily represent those of their affiliated organizations, or those of the publisher, the editors and the reviewers. Any product that may be evaluated in this article, or claim that may be made by its manufacturer, is not guaranteed or endorsed by the publisher.
