# Supplementary material for: Microbial Transformation of Chlordecone and Two Transformation Products Formed During in situ Chemical Reduction
Source: Front Microbiol. 2021 Nov 4;12:742039. doi: 10.3389/fmicb.2021.742039 (PMC8600967; doi:10.3389/fmicb.2021.742039)
Supplement: Supplementary file 1 [file Image_1.pdf]

Supplementary Material

Manuscript: Microbial transformation of chlordecone and two transformation products formed during In Situ Chemical Reduction

Hellal, J.[1*], Saaidi, P.-L.[2], Bristeau, S.[1], Crampon, M.[1], Muselet, D.[2], Della-Negra, O.[2], Mauffret, A.[1], Mouvet, C.[1] et Joulian, C[1].

[1]BRGM, 3 Av Claude Guillemin 45060 Orléans Cedex, France

[2]UMR 8030 Génomique métabolique / CEA / Institut de Biologie François Jacob / Genoscope / Université d'Evry Val d'Essonne / Université Paris-Saclay, France

***Correspondence:** Jennifer Hellal, j.hellal@brgm.fr

Supplementary Figure 1. Examples of chromatogrammes with the detection of either CLD (Top graph) or pentachloroindene (bottom graph) in two samples after 90 days incubation. (1) control without bacteria, (2) with bacteria. 2

Supplementary Figure 2. GC-MS analysis in full scan mode of kinetic experiment with chlordecone (CLD) after 8 months incubation (entry 1 in Figure 2 and in Table S1.). A: incubation with bacteria; B: control incubation (without bacteria). S_8: colloidal sulfur S_8; RT: retention time; MA: manually integrated area. 3

Supplementary Figure 3. GC-MS analysis in full scan mode of kinetic experiment with 10-monohydrochlordecone (-1Cl-CLD) after 8 months incubation (entry 2 in Figure 2 and in Table S1). A: incubation with bacteria; B: control incubation (without bacteria). S_8: colloidal sulfur S_8; RT: retention time; MA: manually integrated area. 4

Supplementary Figure 4. GC-MS analysis in full scan mode of kinetic experiment with trihydrochlordecone (–3Cl-CLD) after 8 months incubation (entry 3 in Figure 2 and in Table S1). A: incubation with bacteria; B: control incubation (without bacteria). S_8: colloidal sulfur S_8; RT: retention time; MA: manually integrated area. 5

Supplementary Figure 5. GC-MS analysis in full scan mode of transfer experiment after 3 months incubation (entries 4 and 5 in Figure 3 and in Table S1). A: incubation of -1Cl-CLD with inoculum from biotic experiment 2 (entry 4 in Figure 2 and in Table S1); B: incubation of -3Cl-CLD with inoculum from biotic experiment 3 (entry 4 in Figure 2 and in Table S1). S_8: colloidal sulfur S_8; RT: retention time; MA: manually integrated area. 6

Supplementary Figure 6. Interpretation of EI mass spectra from GC-MS analysis. A. trihydrochlordecthiol; B. 10-monohydrochlordecthiol; C. chlordecthiol. 7

Supplementary Figure 7. Interpretation of EI mass spectra from GC-MS analysis. A. methyl trihydrochlordecsulfide; B. methyl 10-monohydrochlordecsulfide; C. methyl chlordecsulfide. 8

Supplementary Table S1. Detailed overview of the transformation products resulting from CLD, CLD-1Cl and CLD-3Cl incubations. Values refer to the peak intensity of the most prominent ion of each molecule extracted from the full scan acquisition mode of GC-MS analysis. 12

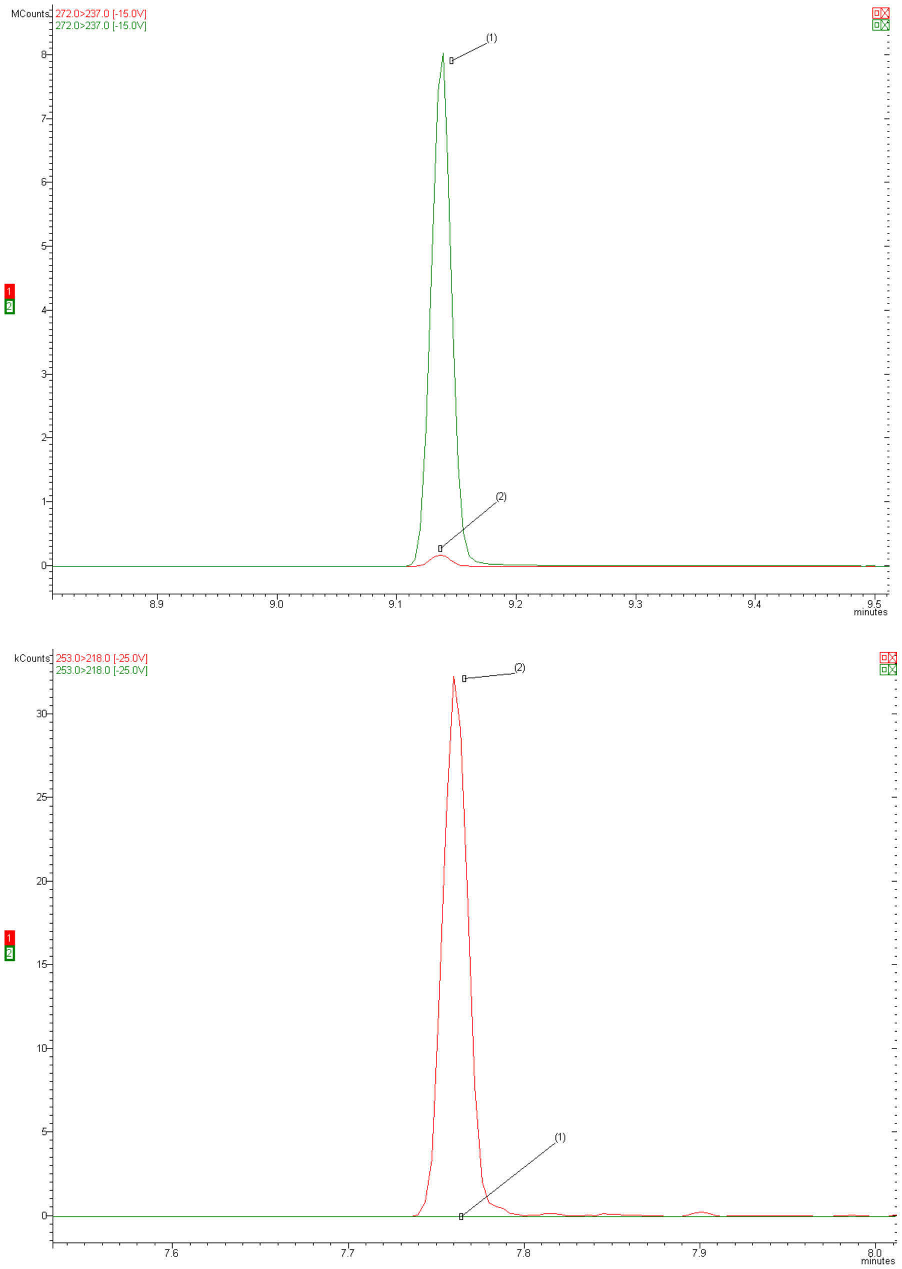

Supplementary Figure 1. Examples of chromatogrammes with the detection of either CLD (Top graph) or pentachloroindene (bottom graph) in two samples after 90 days incubation. (1) control without bacteria, (2) with bacteria.

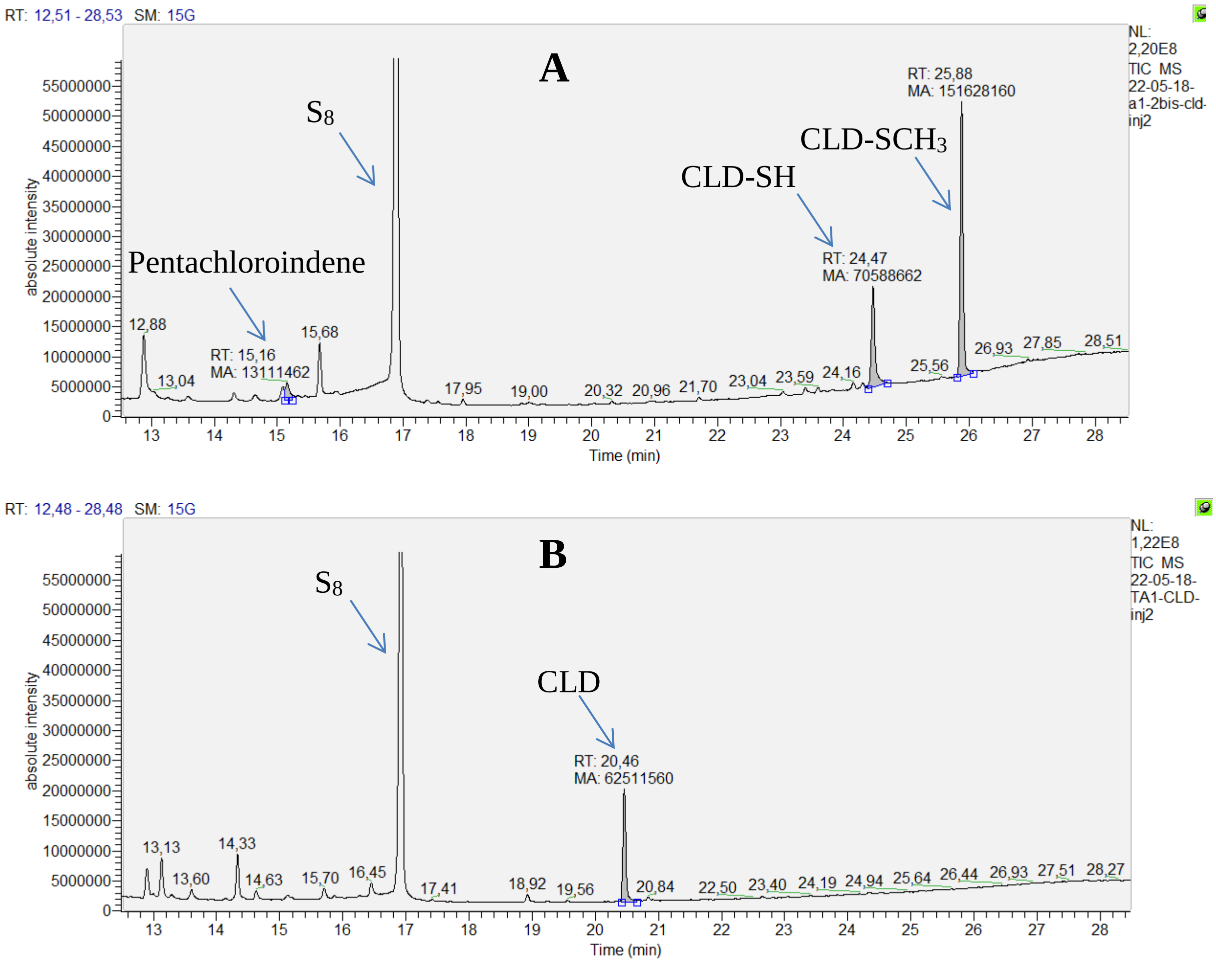

Supplementary Figure 2. GC-MS analysis in full scan mode of kinetic experiment with chlordecone (CLD) after 8 months incubation (entry 1 in Figure 2 and in Table S1.). A: incubation with bacteria; B: control incubation (without bacteria). S_8: colloidal sulfur S_8; RT: retention time; MA: manually integrated area.

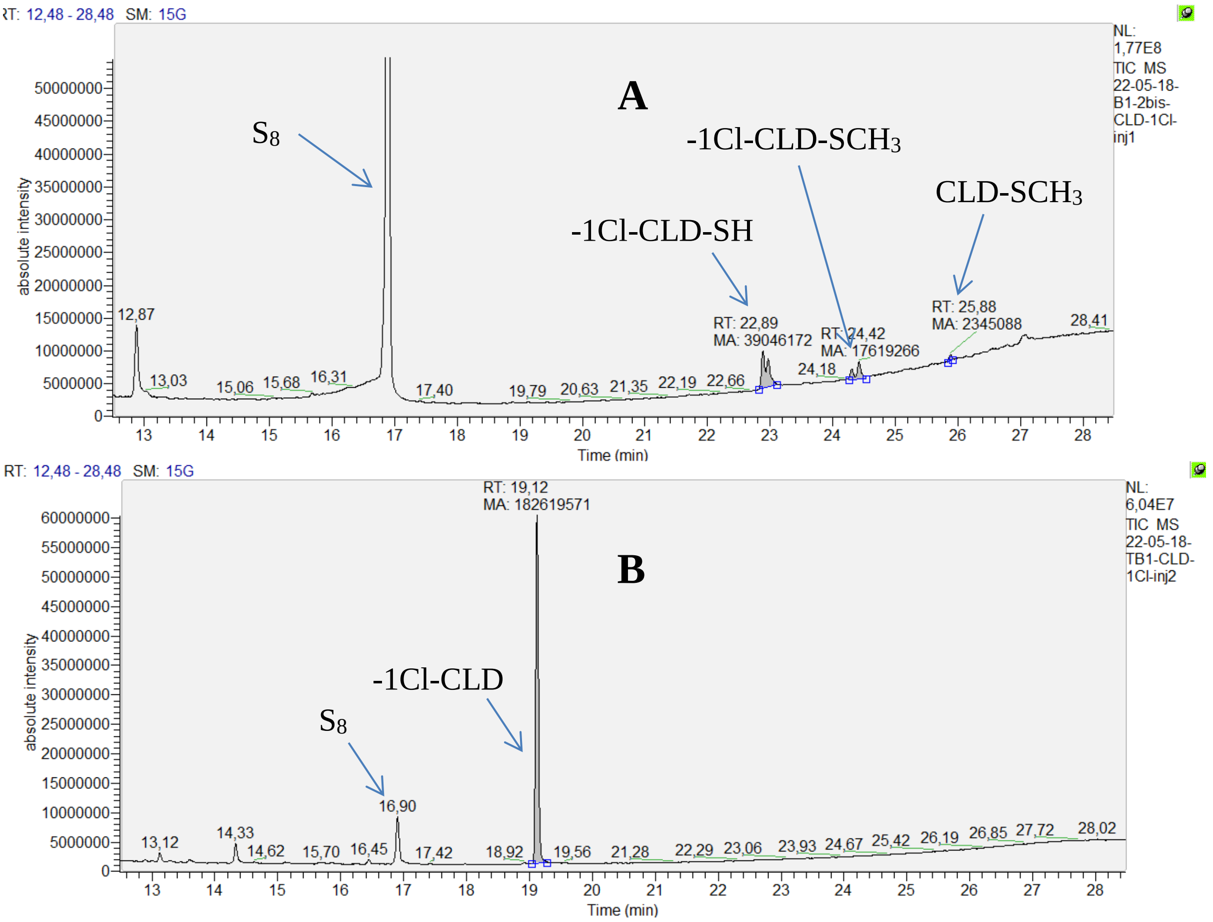

Supplementary Figure 3. GC-MS analysis in full scan mode of kinetic experiment with 10-monohydrochlordecone (-1Cl-CLD) after 8 months incubation (entry 2 in Figure 2 and in Table S1). A: incubation with bacteria; B: control incubation (without bacteria). S_8: colloidal sulfur S_8; RT: retention time; MA: manually integrated area.

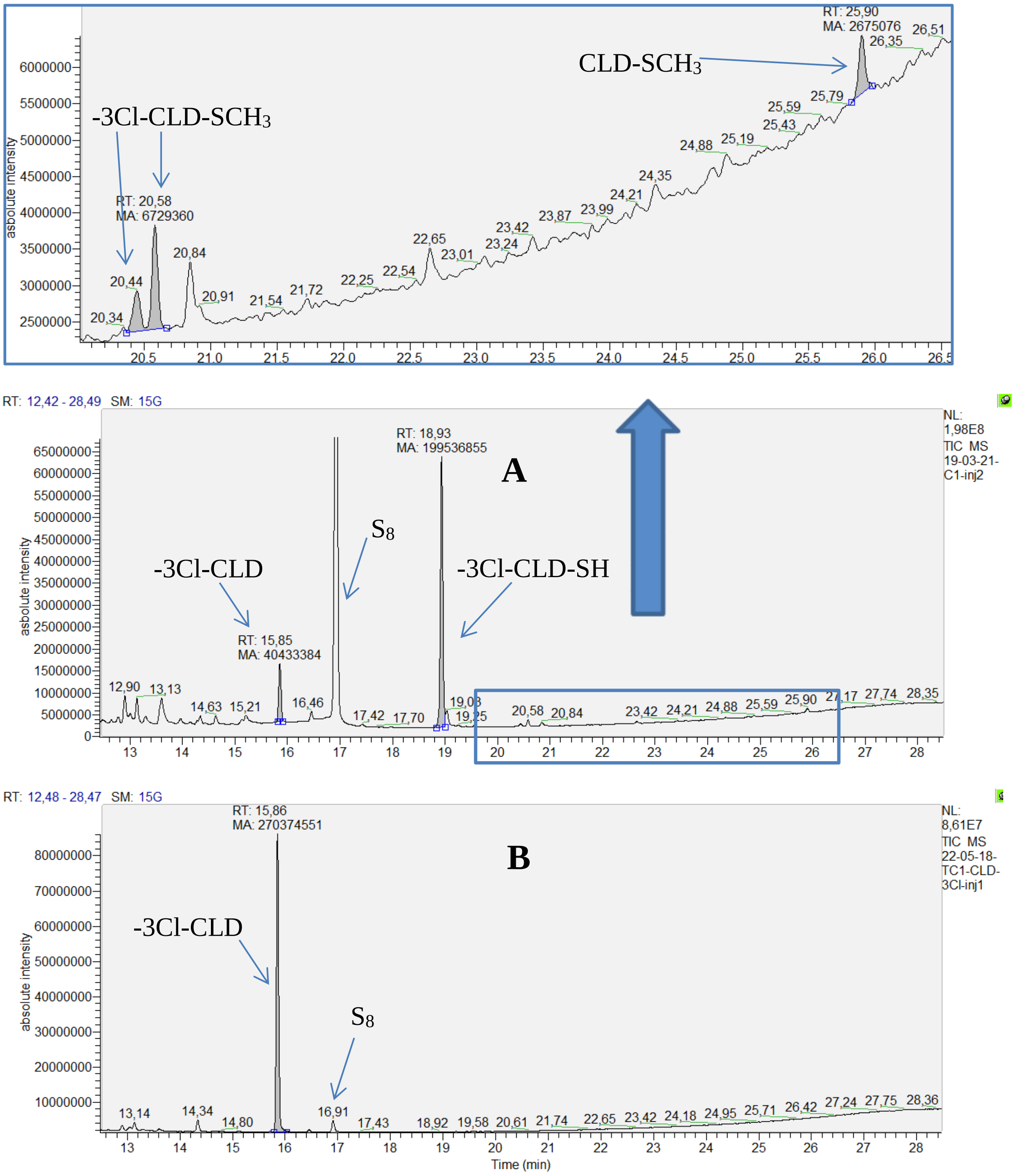

Supplementary Figure 4. GC-MS analysis in full scan mode of kinetic experiment with trihydrochlordecone (–3Cl-CLD) after 8 months incubation (entry 3 in Figure 2 and in Table S1). A: incubation with bacteria; B: control incubation (without bacteria). S_8: colloidal sulfur S_8; RT: retention time; MA: manually integrated area.

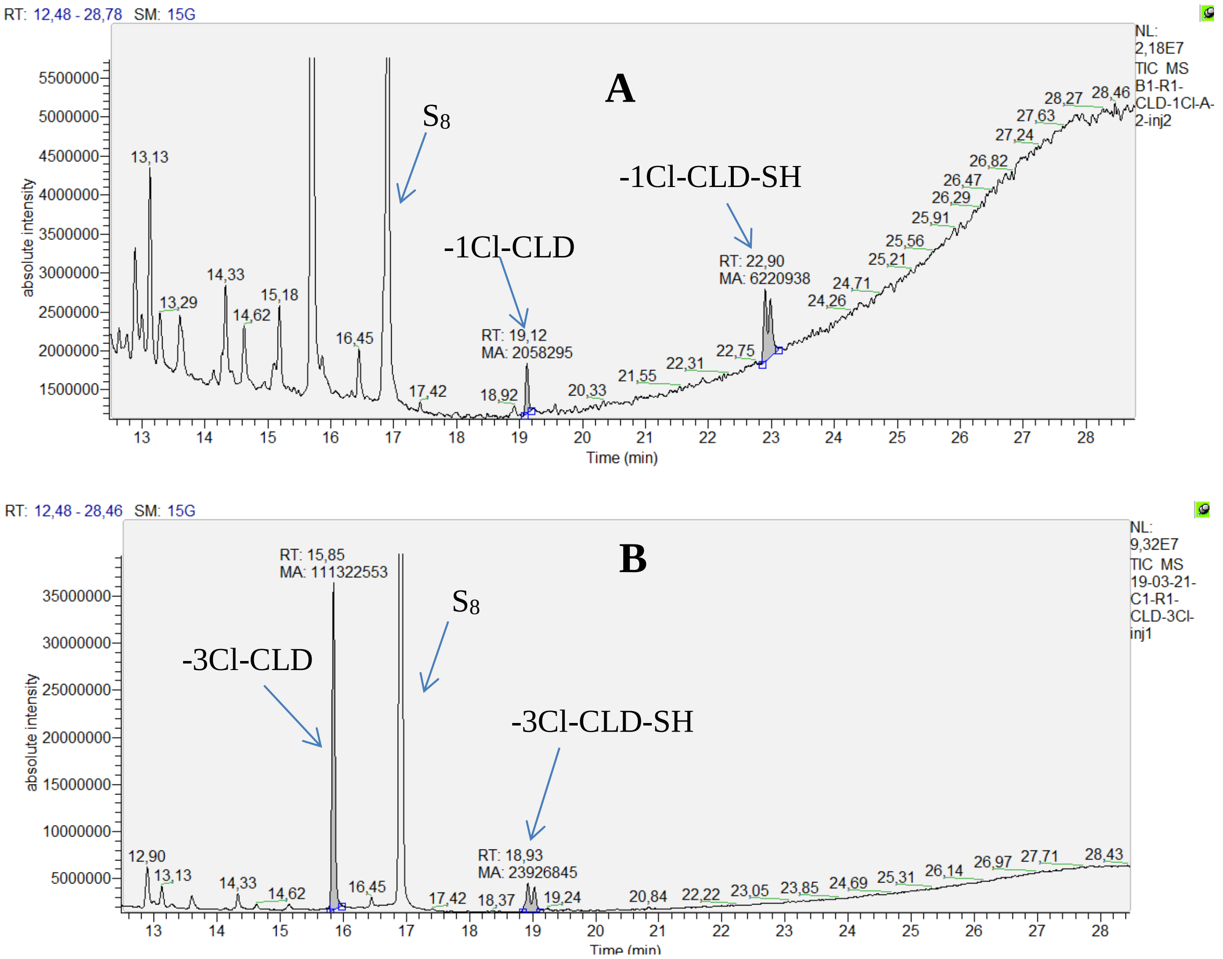

Supplementary Figure 5. GC-MS analysis in full scan mode of transfer experiment after 3 months incubation (entries 4 and 5 in Figure 3 and in Table S1). A: incubation of -1Cl-CLD with inoculum from biotic experiment 2 (entry 4 in Figure 2 and in Table S1); B: incubation of -3Cl-CLD with inoculum from biotic experiment 3 (entry 4 in Figure 2 and in Table S1). S_8: colloidal sulfur S_8; RT: retention time; MA: manually integrated area.

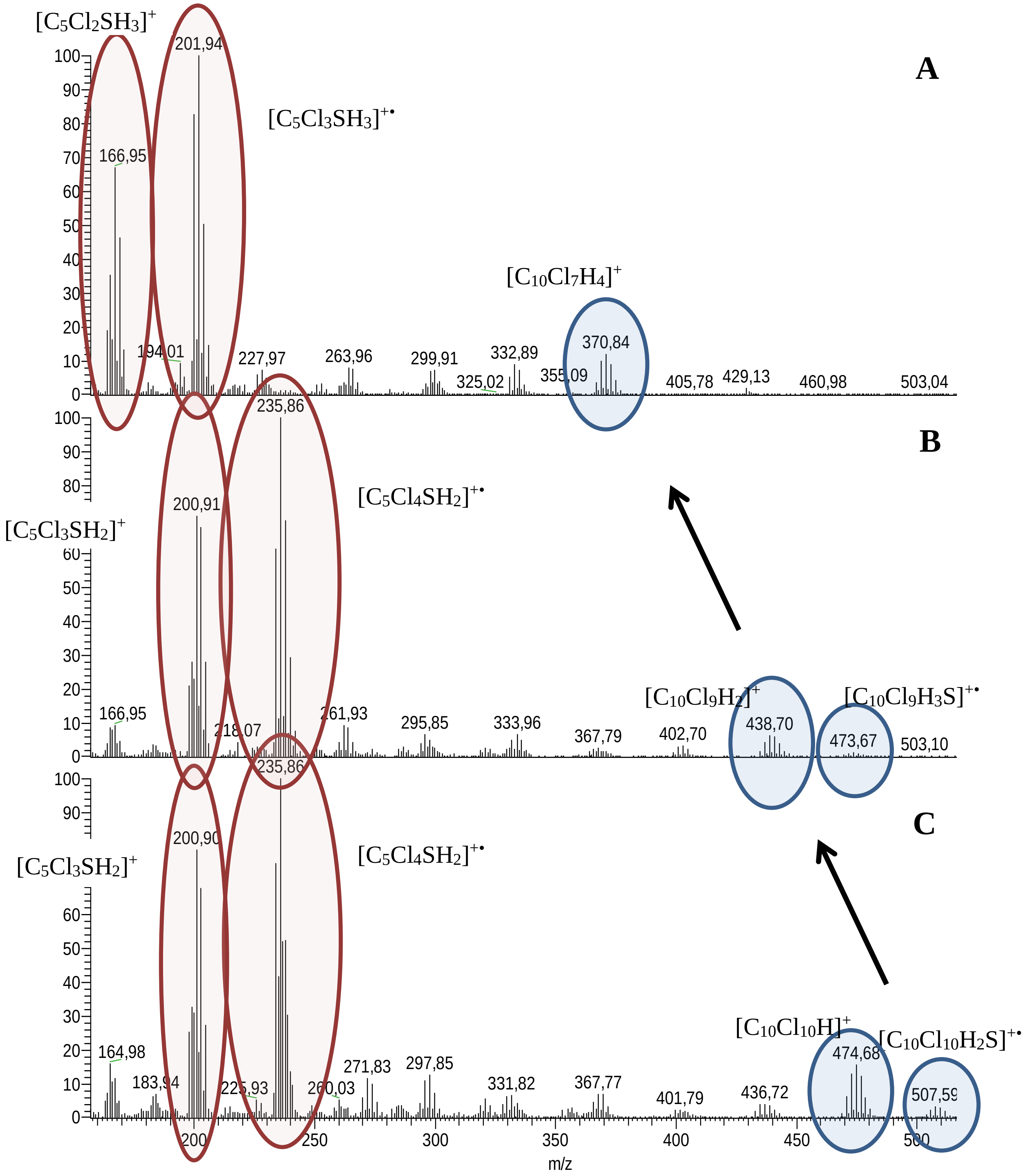

Supplementary Figure 6. Interpretation of EI mass spectra from GC-MS analysis. A. trihydrochlordecthiol (-3Cl-CLD-SH); B. 10-monohydrochlordecthiol (-1Cl-CLD-SH); C. chlordecthiol (CLD-SH).

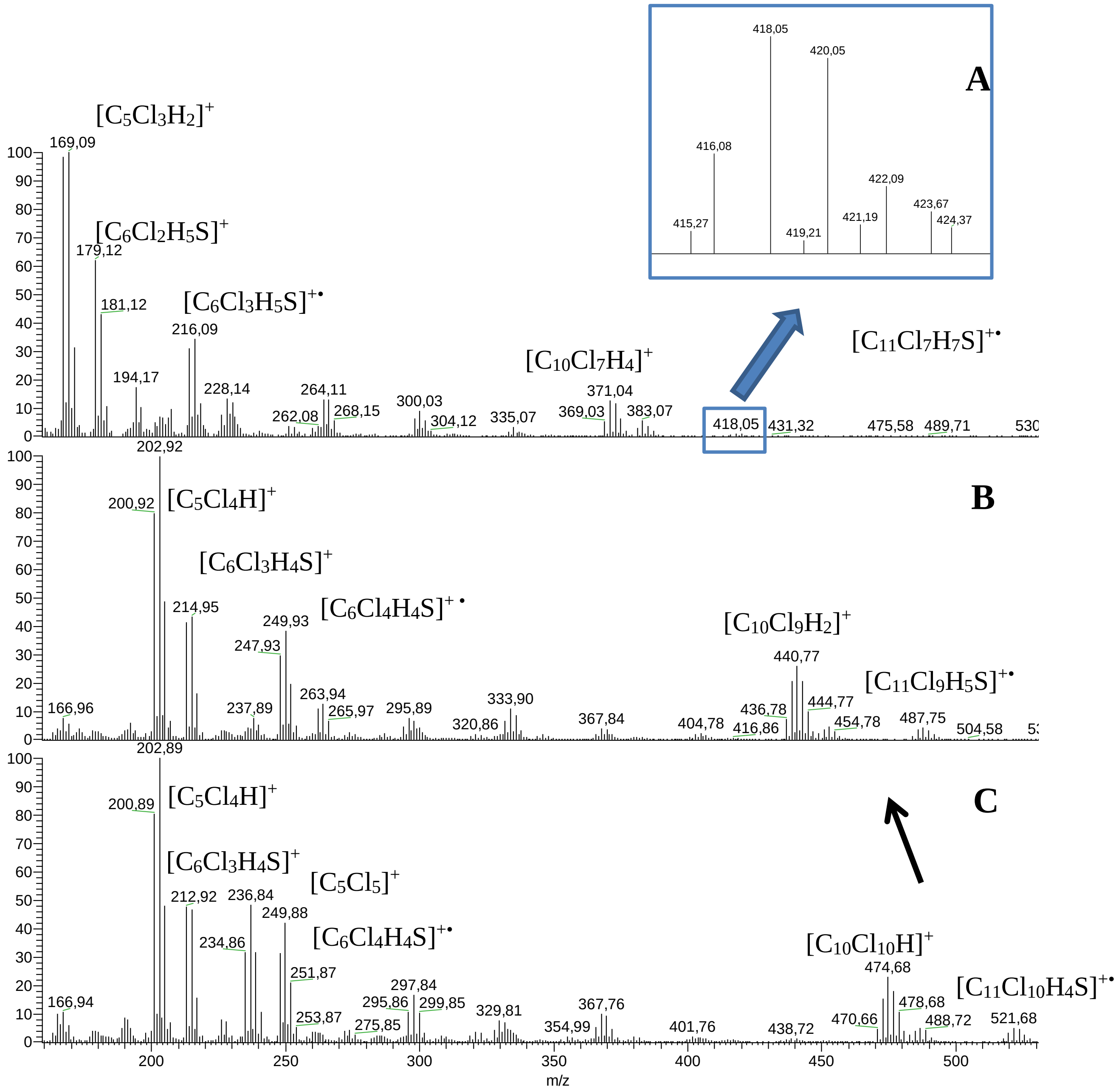

Supplementary Figure 7. Interpretation of EI mass spectra from GC-MS analysis. A. methyl trihydrochlordecsulfide (-3Cl-CLD-SCH_3); B. methyl 10-monohydrochlordecsulfide (-1Cl-CLD-SCH_3); C. methyl chlordecsulfide (CLD-SCH_3).

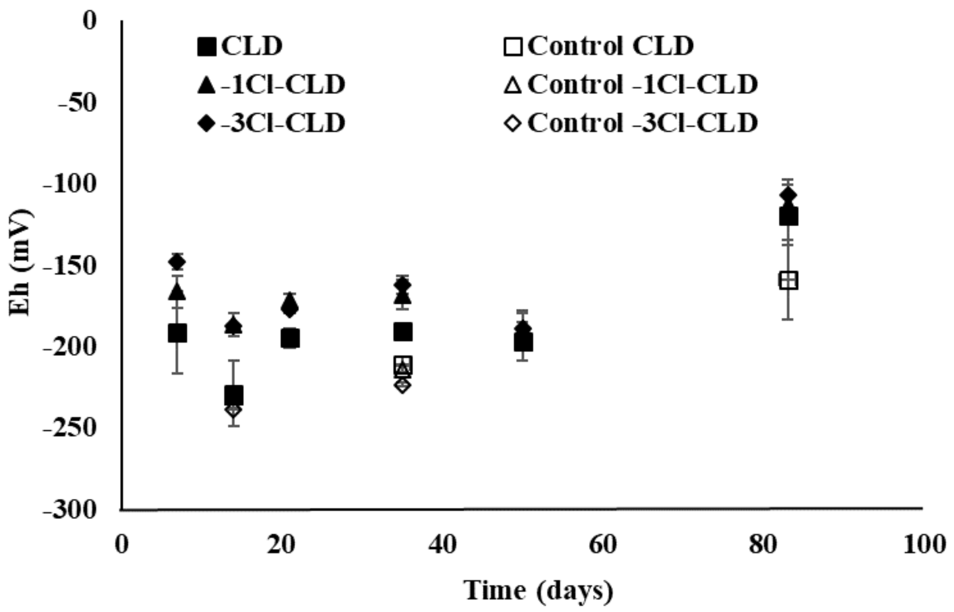

Supplementary Figure 8. Evolution of Eh over time in the incubations

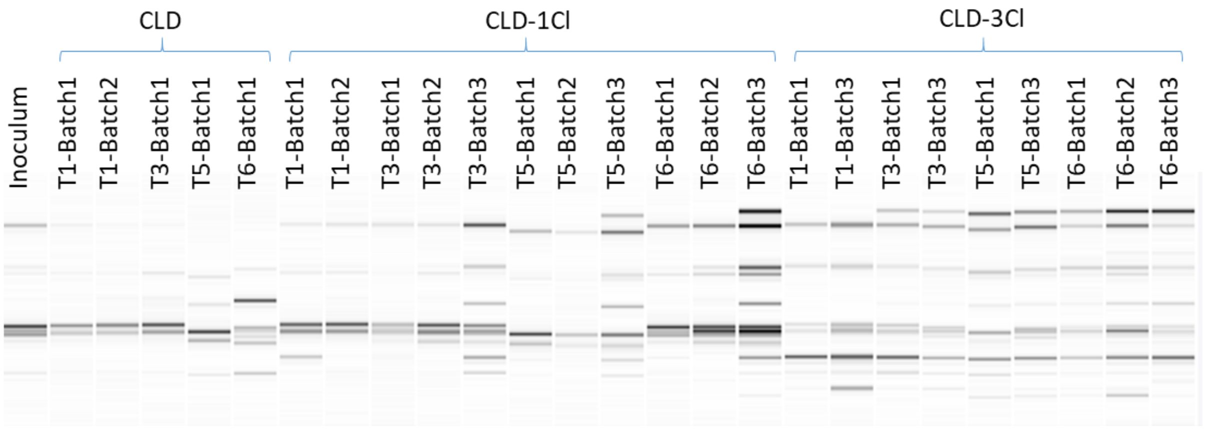

Supplementary Figure 9. Bacterial community CE-SSCP fingerprints in incubations after 8 (T1), 29 (T3), 57 (T5) and 89 (T6) days incubation.

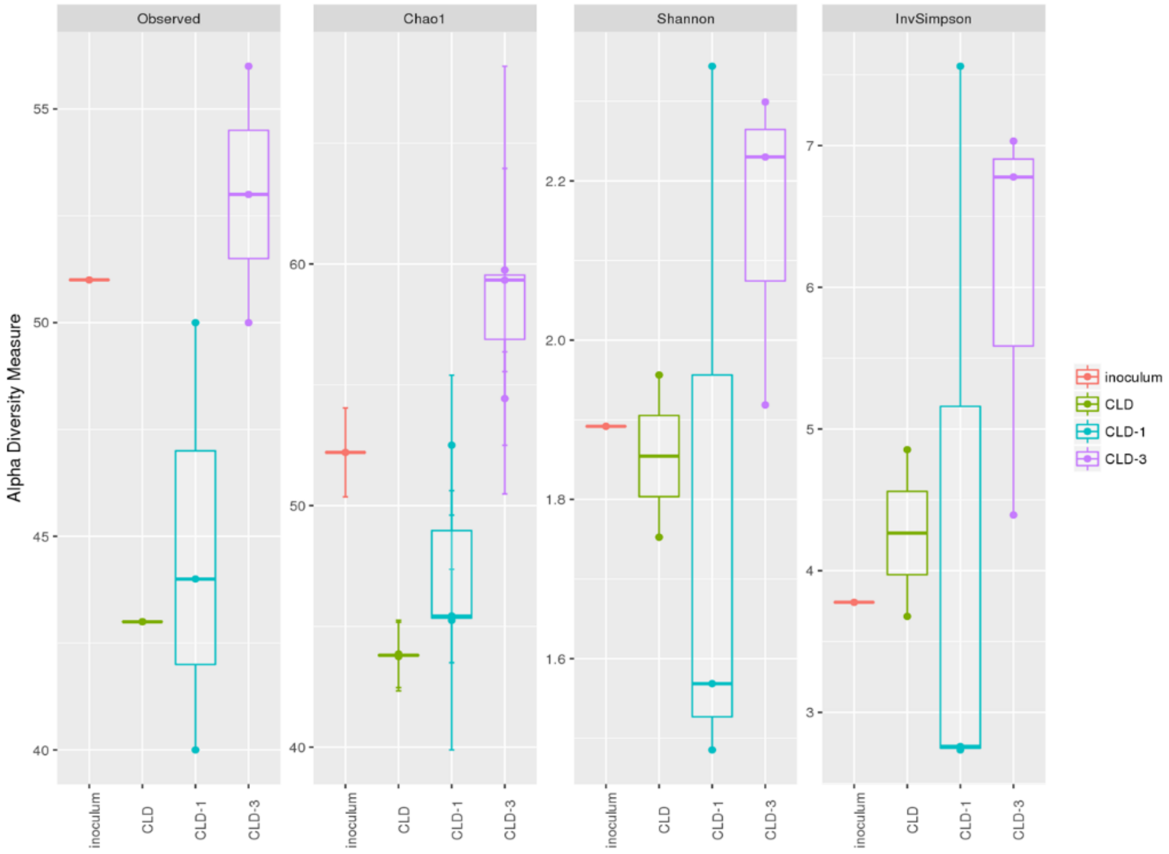

Supplementary Figure 10. Alpha diversity measures.

	compound	CLD	-1Cl-CLD	-3Cl-CLD	pentachloroindene	CLD-SH	-1Cl-CLD-SH	-1Cl-CLD-SH	-3Cl-CLD-SH	-3Cl-CLD-SH	CLD-SCH3	-1Cl-CLD-SCH3	-1Cl-CLD-SCH3	-3Cl-CLD-SCH3	-3Cl-CLD-SCH3
	retention time (GC-MS method 2)	20,46 min	19,12 min	15,85 min	15,16 min	24,47 min	22,89 min	22,97 min	18,93 min	19,03	25,90 min	24,30 min	24,42 min	20,44 min	20,57 min
entry (referred to Fig. 3)	Structure / incubation condition	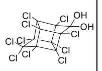	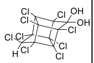	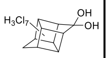	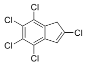	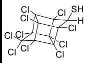	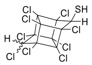	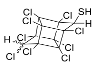	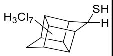	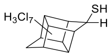	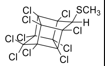	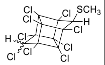	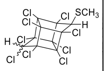	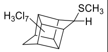	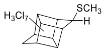
1	CLD with bacteria injection 1				2,11E+06	1,29E+06					2,07E+07				
	CLD with bacteria injection 2				2,45E+06	1,33E+06					2,25E+07				
	CLD without bacteria injection 1	1,02E+07													
	CLD without bacteria injection 2	1,23E+07													
2	-1Cl-CLD with bacteria injection 1						7,66E+06	3,29E+06			4,19E+05	1,37E+06	2,21E+06		
	-1Cl-CLD with bacteria injection 2						4,93E+06	2,31E+06			4,28E+05	1,40E+06	2,14E+06		
	-1Cl-CLD without bacteria injection 1		4,40E+07												
	-1Cl-CLD without bacteria injection 2		5,30E+07												
3	-3Cl-CLD with bacteria injection 1			5,65E+06					2,14E+07	5,74E+05	2,34E+05			2,97E+05	5,15E+05
	-3Cl-CLD with bacteria injection 2			8,85E+06					3,31E+07	8,45E+05	3,86E+05			4,51E+05	7,40E+05
	-3Cl-CLD without bacteria injection 1			5,73E+07											
	-3Cl-CLD without bacteria injection 2			7,86E+07											
4	-1Cl-CLD with bacteria injection 1		5,62E+05				1,14E+06	5,77E+05							
	-1Cl-CLD with bacteria injection 2		5,49E+05				1,19E+06	5,51E+05							
5	-3Cl-CLD with bacteria injection 1			2,34E+07					1,68E+06	7,69E+05					
	-3Cl-CLD with bacteria injection 2			2,44E+07					1,87E+06	8,12E+05					

Supplementary Table S1. Detailed overview of the transformation products resulting from CLD, CLD-1Cl and CLD-3Cl incubations. Values refer to the peak intensity of the most prominent ion of each molecule extracted from the full scan acquisition mode of GC-MS analysis.
